# Natural Feed Additives and Novel Approaches for Healthy Rabbit Breeding

**DOI:** 10.3390/ani12162111

**Published:** 2022-08-17

**Authors:** Iveta Placha, Monika Pogány Simonová, Andrea Lauková

**Affiliations:** Centre of Biosciences of the Slovak Academy of Sciences, Institute of Animal Physiology, Soltesovej 4-6, 040 01 Kosice, Slovakia

Rabbit meat offers excellent nutritive and dietetic properties, but digestive disturbances, mainly during the post-weaning period, induce important economic losses for rabbit farmers. Recently, many studies have focused on feed additives which are able to improve the intestinal health and productivity of broiler rabbits. The main objectives of animal agriculture production are to produce safe food products, eliminating antibiotics with a low impact on environmental pollution. A great deal of interest has been expressed for safe and natural rabbit food with increased nutritional value without inducing bacterial resistance and potential side effects for animals. Natural feed additives such as prebiotics, beneficial microorganisms, organic acids, bacteriocins, and phytogenic compounds are able to match these requirements and can satisfy the increasing consumers’ demand for natural substances; however, because they represent novel valuable substances, their research is an ongoing discipline.

This Special Issue covers a total of eight articles, including seven original studies and one review, with a focus on the effects of natural substances, bioactive compounds, and bacteriocins/enterocins on rabbit production, performance, gastrointestinal microbiota, intestinal immunity and morphology, health parameters, and metabolic processes in rabbits.

Martinez et al. [1] evaluated the effect of *Agave tequilana* stem powder on the growth performance and the intestinal integrity in broiler rabbits, and confirmed the beneficial biological activities of polyphenols and saponins, the main secondary metabolites of this plant. Obtained results justified the utilization of agave powder in rabbit production.

Pogány Simonová et al. [2] tested the preventive effect of a dipeptide enterocin *A*/*P*, produced by *Enterococcus faecium* (Ent) EK13 strain against the methicillin-resistant *Staphylococcus epidermidis* SE P3/Tr2a strain in a rabbit model, determining its effect on the growth performance, phagocytic activity, concentration of secretory immunoglobulin IgA, and gut microbial composition. Good health and increased weight gain reflect the beneficial effect of Ent *A*/*P* on the growth performance of rabbits. The obtained results also showed that the methicillin-resistant *S. epidermidis* SE P3/Tr2a strain did not have any pathogenic effect on rabbits’ health status. The preventive effect of Ent *A*/*P* was recorded due to improved zootechnical parameters, stimulated non-specific immunity, and the stabilized intestinal microbial environment of rabbits.

Roman et al. [3] demonstrated the impact of environmental conditions (laboratory, summer and winter; and outdoor, summer and winter); and dietary supplementation with ethyl esters of linseed oil on the quality of rabbit coat hair. The environmental conditions had a considerable impact on the quality of the rabbit coat; the best results of hair thickness and their heat protection were obtained during the outdoor period. The administration of linseed oil ethyl esters had a positive effect on the hair fatty acids profile; increases in omega-3 acids and decreases in the ratio of omega-6 to omega-3 acids were observed.

Elazab et al. [4] focused their attention on the use of phytogenic feed additives, essential oils (EOs) of rosemary and ginger, as environmentally friendly supplementation to improve rabbit growth performance, feed utilization, meat nutritive value, physiological, and redox status. The authors recommended both EOs for improving sustainable production in the rabbit industry, seeing that body weight gain and feed conversion ratio were improved; cholesterol level in muscle and plasma, as well as triglycerides in plasma, were significantly reduced; muscle fat was decreased; and the oxidant/antioxidant balance was attenuated.

Cremonesi et al. [5] investigated the effect of goji berry, the fruits of the *Lycium barbarum* plant used in traditional Chinese medicine, on the microbiota composition of different parts of the rabbit digestive tract. The obtained results suggested that goji berries could modulate the microbiota by increasing the growth of bacterial families, such as Ruminococcaceae, Lachnospiracae, Lactobacillaceae, and particularly, the genus *Lactobacillus*. To use goji berries as innovative feeds for rabbits, the authors suggested that further studies should evaluate their impact on productive performance, gut immune system maturation, as well as resistance to gastrointestinal disorders.

Bacova et al. [6] showed that to establish suitable concentrations of phytoadditives for a beneficial effect on animal health, metabolic processes of plant compounds in animal organisms should be understood at the molecular level. The metabolic path of thymol, a major constituent of *Thymus vulgaris* L., in the rabbit organism was determined for the first time in their study. The intensive absorption of thymol from the gastrointestinal tract, its metabolism and accumulation in the kidney, and intensive metabolic and excretion processes in the liver were observed. As a consequence of thymol conversion into hydrophilic metabolite and grater elimination in the rabbit organism, thymol was only found in trace amounts in fat and muscle tissues.

Andoni et al. [7] evaluated the effect of goji berry supplementation on the reproductive and productive performance of rabbits. The authors suggested that integration with goji berry in the rabbit diet at 1% affects the reproductive activity, influencing the pattern secretion of luteinizing hormone as well as the sexual receptivity and the productive performance, inducing higher milk production in rabbit does.

A comprehensive review by Placha et al. [8] provides general information on the therapeutic and preventive effect of thymol on various human and animal diseases, followed by its bioavailability in human and animal organisms. Information from this review concerning the mode of action of thymol in animals could also be applied to human medicine and may help in the utilization of herbal medicine in human and veterinary healthcare.

The papers collected in this Special Issue not only present data on the beneficial effects of natural feed additives in rabbit nutrition, but represent the available scientific information regarding the urgent need for more studies to understand the metabolic processes of natural substances on a molecular level, to establish the beneficial dose. The obtained information could be useful for researchers, the veterinary sector, and pharmaceutical industries.
